# Context-Aware Image Compression

**DOI:** 10.1371/journal.pone.0158201

**Published:** 2016-07-01

**Authors:** Jacky C. K. Chan, Ata Mahjoubfar, Claire L. Chen, Bahram Jalali

**Affiliations:** 1 Department of Electrical Engineering, University of California Los Angeles, Los Angeles, California, United States of America; 2 California NanoSystems Institute, Los Angeles, California, United States of America; 3 Department of Bioengineering, University of California Los Angeles, Los Angeles, California, United States of America; Oregon State University, UNITED STATES

## Abstract

We describe a physics-based data compression method inspired by the photonic time stretch wherein information-rich portions of the data are dilated in a process that emulates the effect of group velocity dispersion on temporal signals. With this coding operation, the data can be downsampled at a lower rate than without it. In contrast to previous implementation of the warped stretch compression, here the decoding can be performed without the need of phase recovery. We present rate-distortion analysis and show improvement in PSNR compared to compression via uniform downsampling.

## Introduction

Digital image compression is required for practical storage and transfer of digital images. In addition to conventional photography and online photo sharing applications, it is employed in astronomy [1–3], remote sensing [4–6], machine vision [7,8], digital pathology and radiology [9–11], real-time system monitoring [12,13], and particle-tracking velocimetry [14].

Our prior work has considered the potential for image compression of a process whereby the image is intentionally distorted such that sharp features are self-adaptively expanded before uniform downsampling. The discrete anamorphic stretch transform (DAST) [15] Is inspired by the physics of photonic time stretch, an analog signal processing technique which employs a frequency-dependent all-pass filter with a group delay $\tau (\omega )=\frac{\partial \Phi (\omega )}{\partial \omega }$ to slow down fast analog temporal waveforms so they can be digitized in real time [16–19]. By doing so, photonic time stretch has led to the discovery of optical rogue waves [20], the creation of a new imaging modality known as the time stretch camera [21], which has enabled detection of cancer cells in blood with record sensitivity [11] and a portfolio of other fast real-time measurements, such as an ultrafast vibrometer [22], the discovery of soliton explosions [23] and the observation of relativistic electron structures [24]. While time stretch slows down the fast time series so it can be digitized in real-time, it conserves the time-bandwidth product. Recently, it has been shown that this product can be reduced or expanded for the information carried by the signal envelope, leading to time-bandwidth engineering [25]. This in turn has led to the concept of the “information gearbox”, as well as photonic hardware accelerators, for real-time data acquisition, analytics and high performance computing [26].

In DAST, two dimensional discrete spatial coordinates[*x*,*y*], replace one dimensional time coordinate *t*. A warped version, $\overset{˘}{E}[x,y]$, of the input image, *E*[*x*,*y*], is generated by applying a warp kernel $\tilde{K}[u,v]=\exp\;j\Phi [u,v]$ to the input spectrum $\tilde{E}[u,v]=FFT^{2}\{E[x,y]\}$ in the following transformation:

$$\overset{˘}{E}[x,y]=\left|IFFT^{2}\{\tilde{E}[u,v]\cdot \tilde{K}[u,v]\}\right|,$$ Eq 1

where *IFFT*^2^ is the two-dimensional discrete inverse Fourier transform operator. The warping kernel preferentially stretches the sharp features so that it can be downsampled at rates much lower than what was previously possible with naïve uniform downsampling. This property is conducive to image compression and its digital implementation has also led to a new powerful edge detection algorithm for extracting features from digital images [27].

As seen in Eq (1), the warped mapping is performed in the frequency domain. Image reconstruction then requires knowledge of the phase of the transformed image. In the previous work, ideal phase recovery was assumed to show the potential of warped stretch for data compression [15]. The accuracy of phase recovery from intensity (brightness) data is subject to the signal to noise ratio (SNR) [28–34] and this limits the fidelity of image reconstruction from de-warping [35].

Here, we report a related approach to warped stretch that does not require phase recovery. This physics-inspired numerical algorithm is inspired by the recent report of the first demonstration of analog image compression [36]. In this technique, a laser pulse is spread out spatially into a one-dimensional rainbow by a diffraction grating, then incident onto a single line of the input sample. Pixels of the to-be-compressed image are therefore mapped onto this incident laser pulse rainbow as intensity changes in each wavelength along the 1D rainbow. The pulse is then time stretched with a fiber Bragg grating with a highly nonlinear group delay profile that imparts warped mapping of spectrum (space) into time. The resulting temporal signal is digitized followed by reconstruction by performing inverse mapping numerically. Similar to the previous compression method, the non-linear dispersion relation of the optical fiber functions as the warp kernel. Since the image information is mapped directly onto the signal spectrum, no phase recovery is needed.

We are inspired to implement this process in a numerical algorithm and investigate its utility for application to digital image compression. In this technique, the input signal is directly warped then uniformly sampled. This two-step process achieves context-aware non-uniform sampling but without the need for Fourier transformation and phase recovery. With proper design of the warp kernel [37], sharp spatial features are stretched much more than slow-varying ones in a context-aware manner. The redistribution of the local signal entropy lowers the overall Nyquist sampling rate and is the basis of the “information gearbox” concept proposed in [26].

## Methods

**Fig 1:**
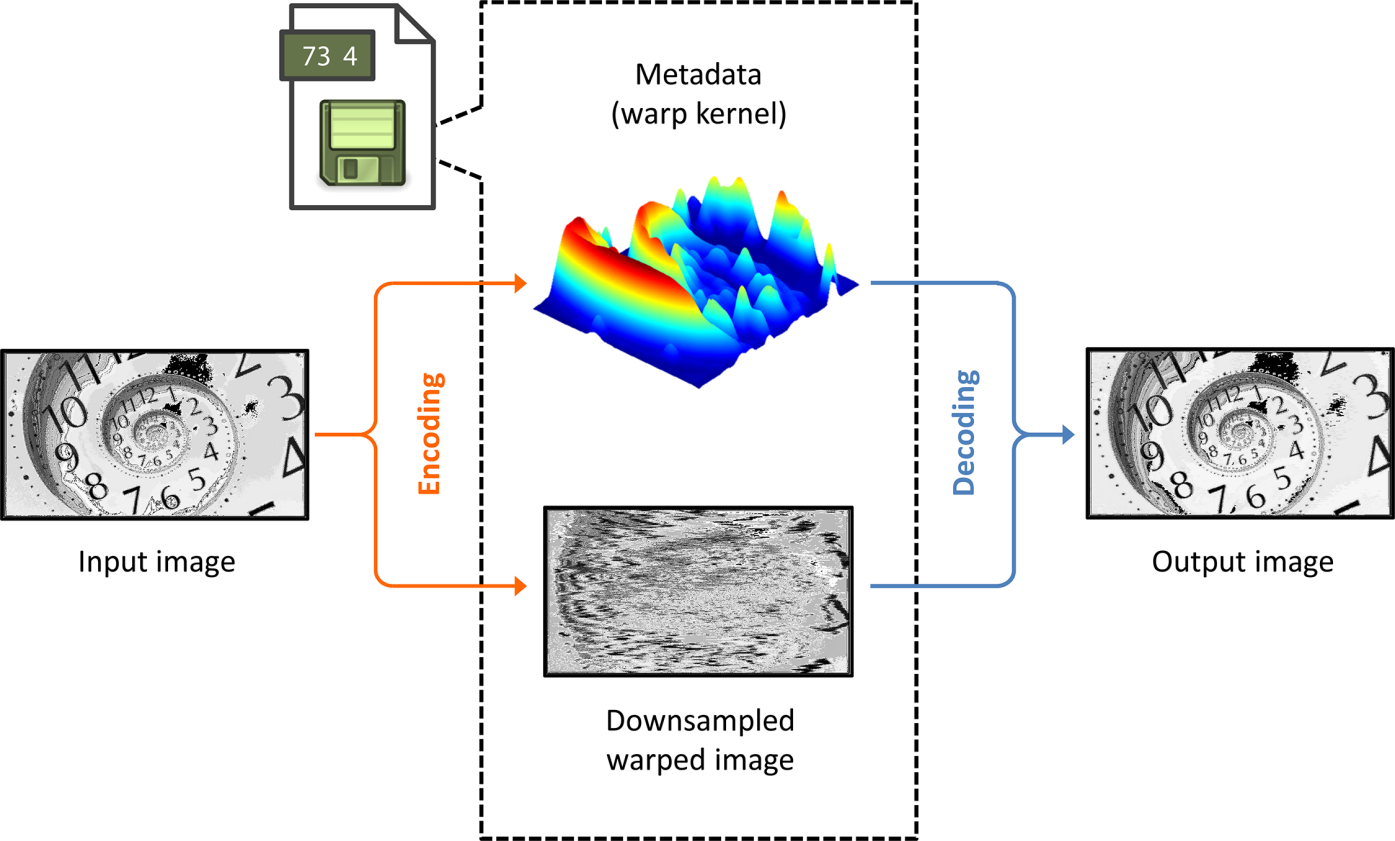
Overview schematic for image compression codec with warped stretch. The input is split into two components: i) the downsampled warped image and ii) the metadata, which contains a compressed version of the warp kernel. These two components are jointly used for recovering the original input. Since the warp kernel is image-dependent, we must send it as part of the compressed file, which creates extra overhead relative to an image-independent compression technique, such as uniform sampling. However, if the metadata can be compressed extremely compactly, the overall compression ratio can still be significant.

Fig 1 illustrates how the direct warped stretch transform can be used as a compression codec. We consider signal-dependent warp kernels that are specifically tailored to the spatial sparsity patterns in the input signal for optimal non-uniform sampling. Since the warp kernel is signal-dependent, it must also be sent along with the downsampled image as reconstruction metadata. The compressed image can then be recovered by de-warping and upsampling.

Traditional non-uniform sampling avoids the overhead associated with the metadata by considering irregularly spaced samples in 2D space (such as 2D contours [38,39]), by requiring strong statistical assumptions on the input [40], or by ignoring the need to know the positions of the non-uniform samples [41]. The overhead associated with knowing the sample positions in conventional non-uniform sampling is equivalent to the metadata that contains the warp kernel in our approach.

In traditional non-uniform sampling, the information about sample position is often non-sparse and thus the overall compression from non-uniformly sampling will be limited by the compressibility of the sample position metadata. This important problem is mitigated in our approach because the warp kernel only specifies the recommended local sampling density of the image, not the exact sampling locations. This results in a more compact metadata albeit at the cost of a marginally lower SNR.

Additionally, separating the warping information from both the downsampling stage and the exact image pixel locations frees us to re-use the warp kernel elsewhere. For example, in the situation where one wants to compress the same file at a different quality, the same warp kernel design can be re-used at different downsampling rates (i.e. compression ratios), thus reducing the overall pre-processing burden. Alternatively, one can consider simultaneously compressing any set of inputs that share similar sparsity statistics (e.g. different colour channels, passport photo libraries) together, by generating a single warp kernel tailored to warp the “average” of the input set.

**Fig 2:**
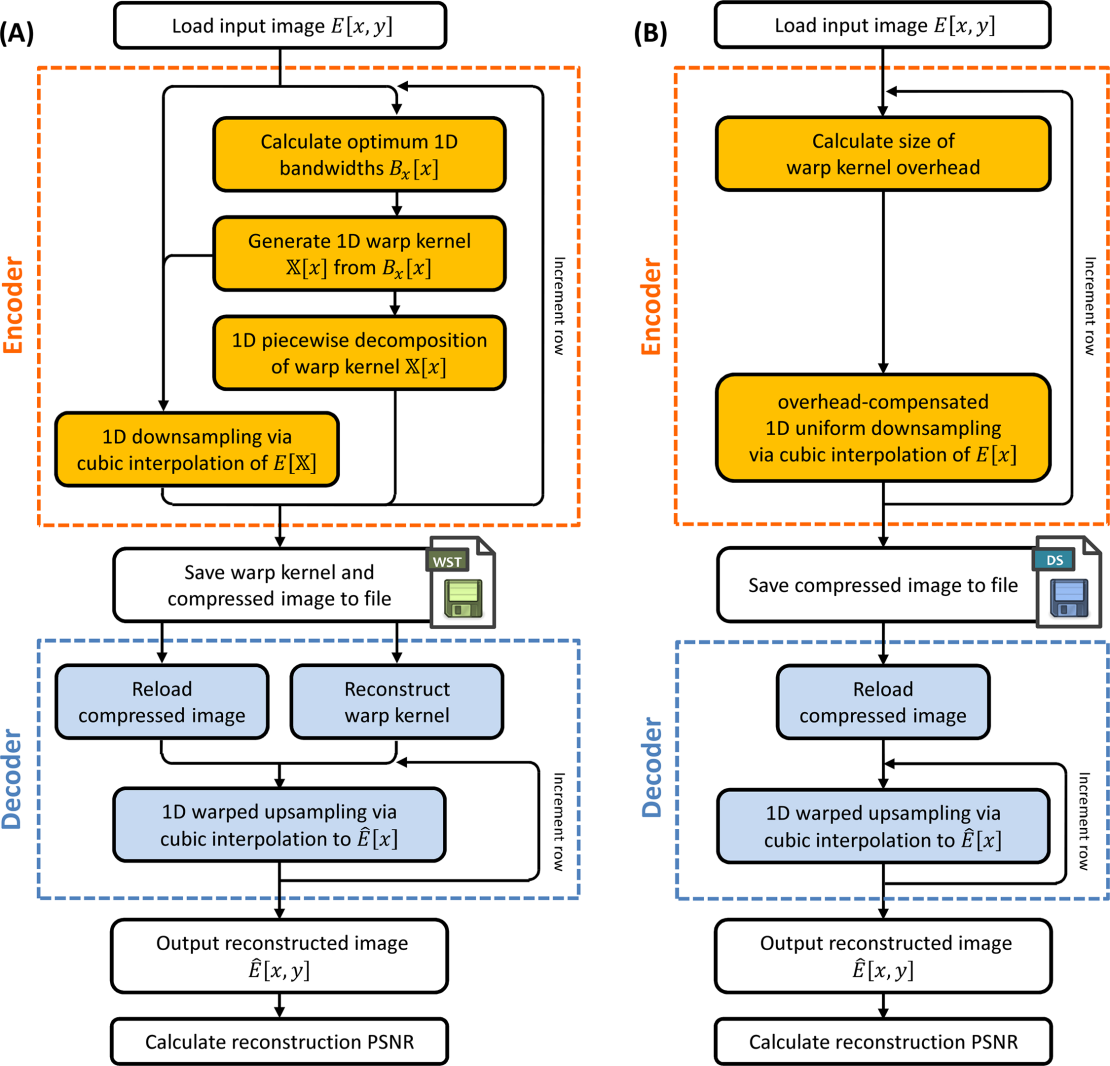
Flowchart for warped stretch compression using piecewise decomposition. (A) A separate warp kernel is generated for each row of the image. The warp is based on the local row bandwidth, i.e. the derivative of the image intensity. The input image is then warped by the kernel and downsampled at a uniform rate. The compressed image is then saved into a custom binary file format (WST), along with the warp kernel, which itself is compressed via piecewise decomposition. To reconstruct the image, we decompress the piecewise-decomposed warp kernel and use it to perform non-uniform upsampling on the reloaded compressed image. (B) For comparison purposes, we also uniformly downsample the input image in 1D with a lower downsampling rate that accounts for the warp kernel overhead saved in the WST binary format.

As a proof-of-concept demonstration, we show the performance of warped stretch compression using piecewise decomposition of the warp kernel. Fig 2 shows this implementation. We emphasize that this is just one instantiation of the general compression scheme shown in Fig 2; other implementation schemes are possible with potentially better performance.

The optimal warp kernel is designed according to the methodology adapted from [37]. In [37], the short-time Fourier transform (STFT) was employed to evaluate the relative local bandwidth *B*_*ω*_(*ω*) of the input spectrum (called the “frequency of spectrum”). When hardware bandwidth limitations do not exist, the required bandwidth to capture the stretched signal can be expressed as:

$$B_{\omega }(\omega )=\frac{1}{4\sqrt{\pi }}\sqrt{\left|\frac{d\tau_{g}(\omega )}{d\omega }\right|}$$ Eq 2

This relates the ideal 1D warp kernel *τ*_*g*_(*ω*) to the local bandwidth via its derivative:

$$\tau_{g}(\omega )=\int |\frac{\partial \tau_{g}(\omega )}{\partial \omega }|d\omega$$ Eq 3

The method in [36,37] guarantees a warp kernel that is ideal for minimizing the time-bandwidth product of the signal envelope in 1D. In terms of compression, the ideal warping kernel for image compression should minimize the Nyquist sampling rate after stretching. Given a discrete bandwidth measure *B*_*x*_[*x*] and barring any additional constraints imposed by the compression itself (e.g. kernel overhead constraints), the ideal sampling locations after warping X[x] are those which reshape the bandwidth of the warped output $B_{X}[X]$ to be as close as possible to the average original bandwidth ⟨*B*_*x*_[*x*]⟩ across the image row:

$${\arg\;\min}_{X}\;{\left\|B_{X}[X]-\langle B_{x}[x]\rangle \right\|}_{1}$$ Eq 4

We will limit our consideration to kernels that provide ideal one-to-one warp mapping, as defined by the warp kernel $\tau_{g}=X[x]$. Note that while we have borrowed the mathematical notation used in [36,37,42], here original and warped sampling locations *x* and X[x] both denote pixel locations and no longer carry physical meaning. A kernel bandwidth measure $B_{X}[X]$ that simultaneously minimizes Eq (4) and provides one-to-one mapping is possible for an STFT window size of 2 pixels, in which case the STFT reduces to the magnitude of the discrete signal derivative $\left|\frac{\Delta \tilde{E}}{\Delta X}\right|$:

$$B_{X}[X]=\left|\frac{\Delta \tilde{E}[x]}{\Delta X[x]}\right|=\left|\frac{\Delta \tilde{E}[x]}{\Delta x}\right|\;{\left|\frac{\Delta X[x]}{\Delta x}\right|}^{-1},$$ Eq 5

where Δ*x* = *x*_*i*_ − *x*_*i*−1_ is the original (uniform) sample spacing, and $\Delta X[x_{i}]=X[x_{i}]-X[x_{i-1}]$ is the non-uniform sample spacing. Due to the differing objectives of optimization, we see that the derivative of the kernel is now inversely proportional to the bandwidth measure, instead of having a quadratic dependence, as shown in Eq (2). Using the same bandwidth measure for the original signal, i.e. the magnitude of the signal derivative, we find that the expression in Eq (4) can be minimized with:

$$B_{X}[X]=\langle B_{x}[x]\rangle ≜\left\langle |\frac{\Delta \tilde{E}[x]}{\Delta x}|\right\rangle$$ Eq 6

Using Eqs (3), (5) and (6), we then arrive at our expression for the optimal kernel:

$$X[x]=\sum_{i}|\frac{\Delta \tilde{E}[x_{i}]}{\Delta x}|\frac{\left|\Delta x\right|}{\left\langle B_{x}[x]\right\rangle }=\sum_{i}\frac{\left|\Delta \tilde{E}[x_{i}]\right|}{\left\langle \frac{\left|\Delta \tilde{E}[x]\right|}{\left|\Delta x\right|}\right\rangle }$$ Eq 7

To compress the input image, the kernel is applied via cubic interpolation, and the warped output is uniformly downsampled. Because of the redistribution in local entropy, this downsampling rate is in general greater than a uniform downsampling rate that achieves the same resultant image quality. It should also be noted that Eq (6) implies that images with high average bandwidth ⟨*B*_*x*_[*x*]⟩ will also have limited compressibility.

Representing the metadata, the kernel itself is compressed via piecewise decomposition: compression is achieved by saving only the positions of the turning points of each kernel row, which are identified by placing an energy threshold on the discrete derivative of the local bandwidth *B*_*x*_[*x*]. Alternatively, STFT with a larger window size can be used, but this will be computationally more expensive.

As an initial demonstration, we used an 8-bit 1672×2800-pixel image of a grayscale fractal clock and performed compression on each row. The compressed image (saved with 8-bit precision) was saved along with the metadata kernel (saved in single precision) in a custom binary file format, identified with extension WST. To reconstruct the input image, the sampling positions of the compressed image were regenerated by the piecewise-decomposed warp kernel by cubic interpolation, then used to perform non-uniform upsampling on the reloaded compressed image (again by cubic interpolation).

To benchmark our results, we created a similar binary file containing the uniformly downsampled image (saved at a bit depth of 8 bits). To normalize our compression performance over file size, we decreased the uniform downsampling rate such that the resultant file size was equal to the file size of the WST binary file, which contains the metadata kernel in addition to the compressed image. The reconstructed images were then compared in terms of the peak signal-to-noise ratio (PSNR) relative to the original input image.

## Results and Discussion

**Fig 3:**
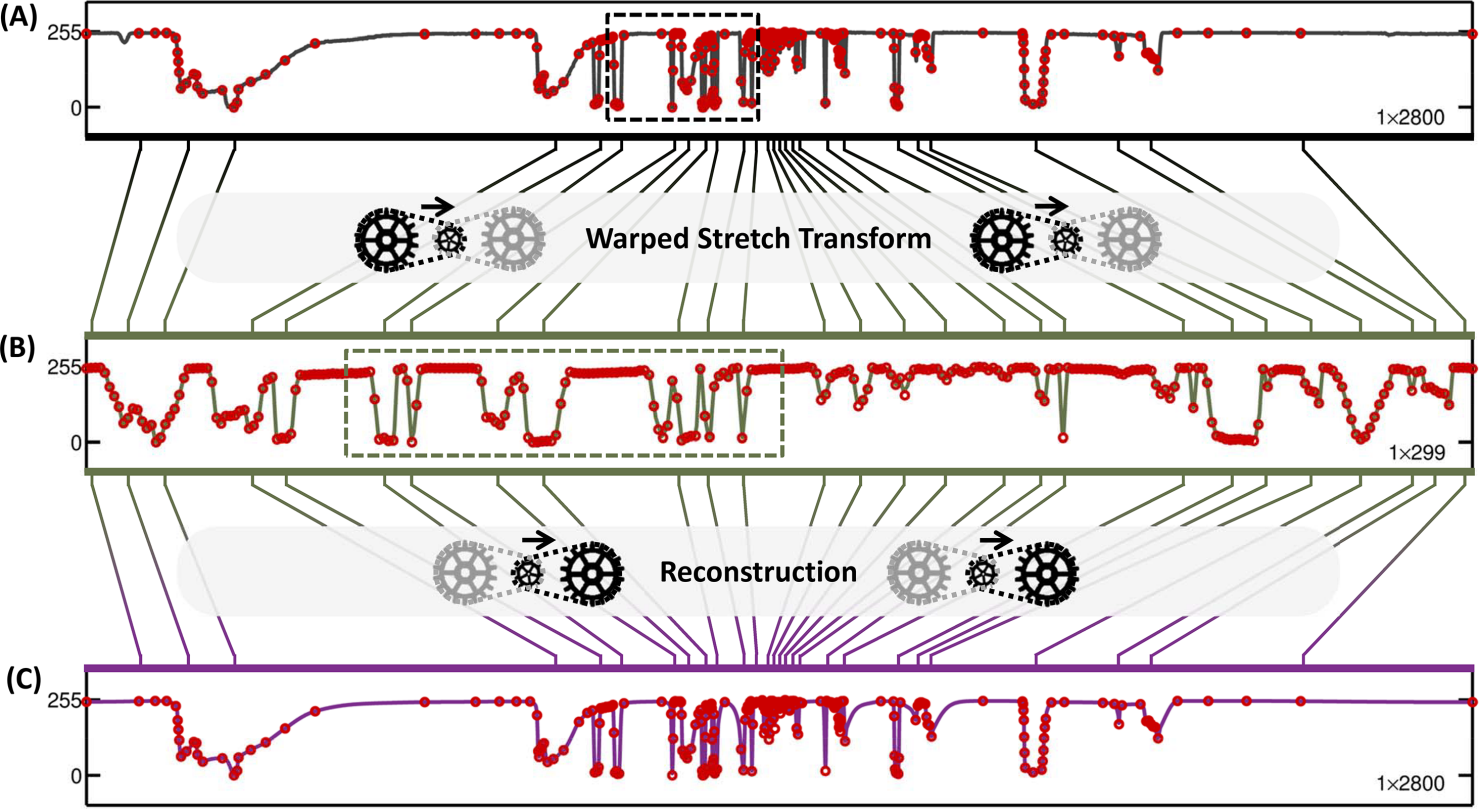
Results for row 838 (out of 1672) of the fractal clock image at 6X warped stretch compression with overhead compensation. The line signal (A) is first rescaled using non-uniform cubic interpolation as defined by the warp kernel, generated according to Eq (7). In this warped space (B), the signal can now be downsampled at a uniform rate (indicated by the red circles) that is lower than what is possible using uniform downsampling, at a given reconstruction quality. The number of downsampled points is less than 1/8^th^ of the number of pixels in the original line signal so that the compression ratio becomes 8X after taking the warp kernel overhead into consideration when saving to file. Both the warping and the downsampling operations can be reversed to reconstruct the line signal (C). The corresponding locations of the downsampled points (red circles in (B)) overlay the (A) original and (C) reconstructed line signals for visual reference. The dashed frames in (A) and (B) are shown in closeup form in Fig 4.

Fig 3 shows the warping / de-warping process for one row of the fractal clock image at 6X compression. The waveform is first rescaled using non-uniform cubic interpolation as defined by the warp kernel generated for this row. In this warped state, the signal can now be downsampled at a uniform rate that is lower than what is possible using uniform downsampling, with equivalent reconstruction quality. Both the warping and the downsampling operations can be reversed to reconstruct the original line signal, and can be seen as abstractions of the analog spectrotemporal reshaping operations, fulfilling the role of an information gearbox [26].

**Fig 4:**
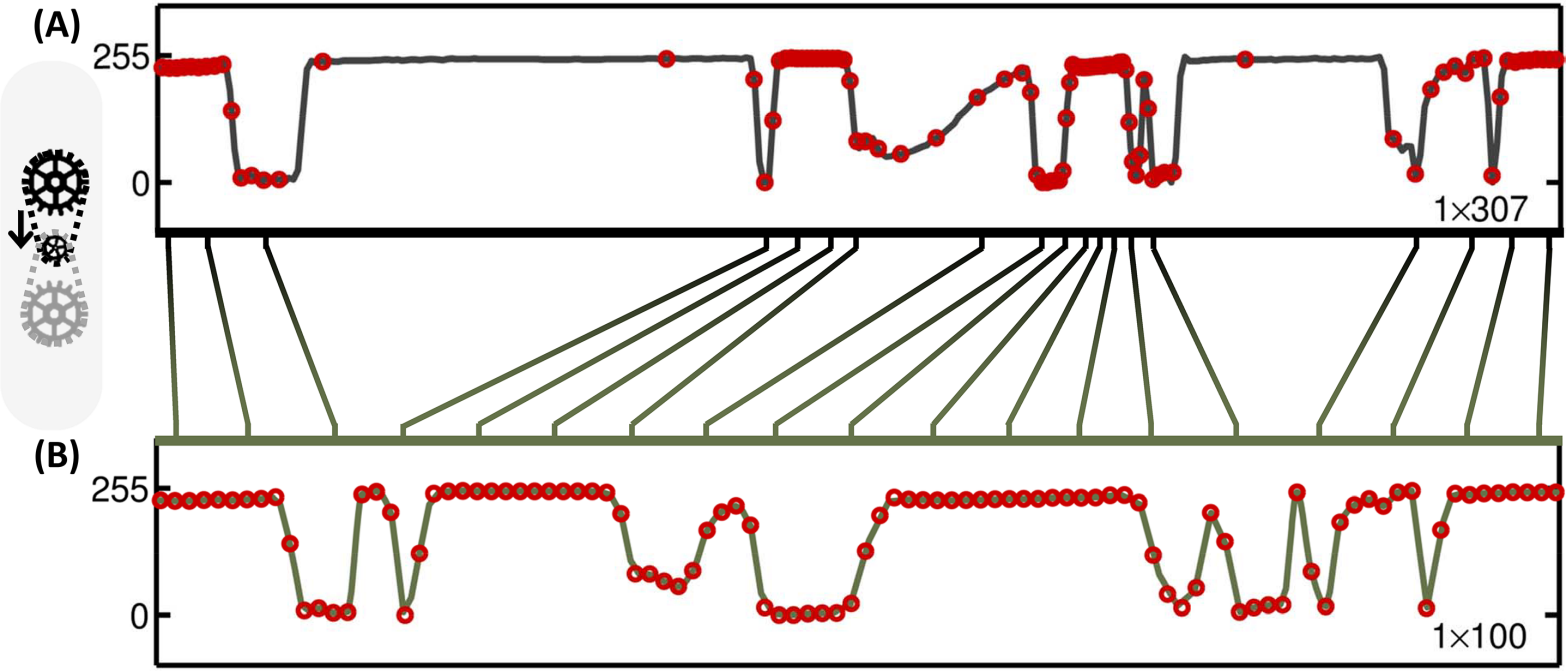
Closeup of Fig 3A and 3B. (A) A particular subsection of row 838 of the fractal clock image (dashed frame in Fig 3A), which contains a mixture of feature-sparse and feature-dense regions, is shown in expanded form. (B) The same subsection, after warping by the warp kernel (dashed frame in Fig 3B). The subsection in (B) matches the length of the original line subsection (A) to show the redistribution of the feature density caused by the warped stretch transform. The corresponding locations of the downsampled points (red circles in (B)) overlay the original line signal (B) for visual reference.

In principle, the warping process redistributes the signal, such that the local entropy becomes uniformly distributed as shown in Fig 4. However, as mentioned in the previous section, the ideal warp kernel is overly complex (i.e. as complex as the input itself) and thus merits compression. In the present case, this was achieved by piecewise decomposition. Compressing the warp kernel results in some loss of information in the overall compression; this is reflected in the non-ideal allocation of points in Fig 4B.

**Fig 5:**
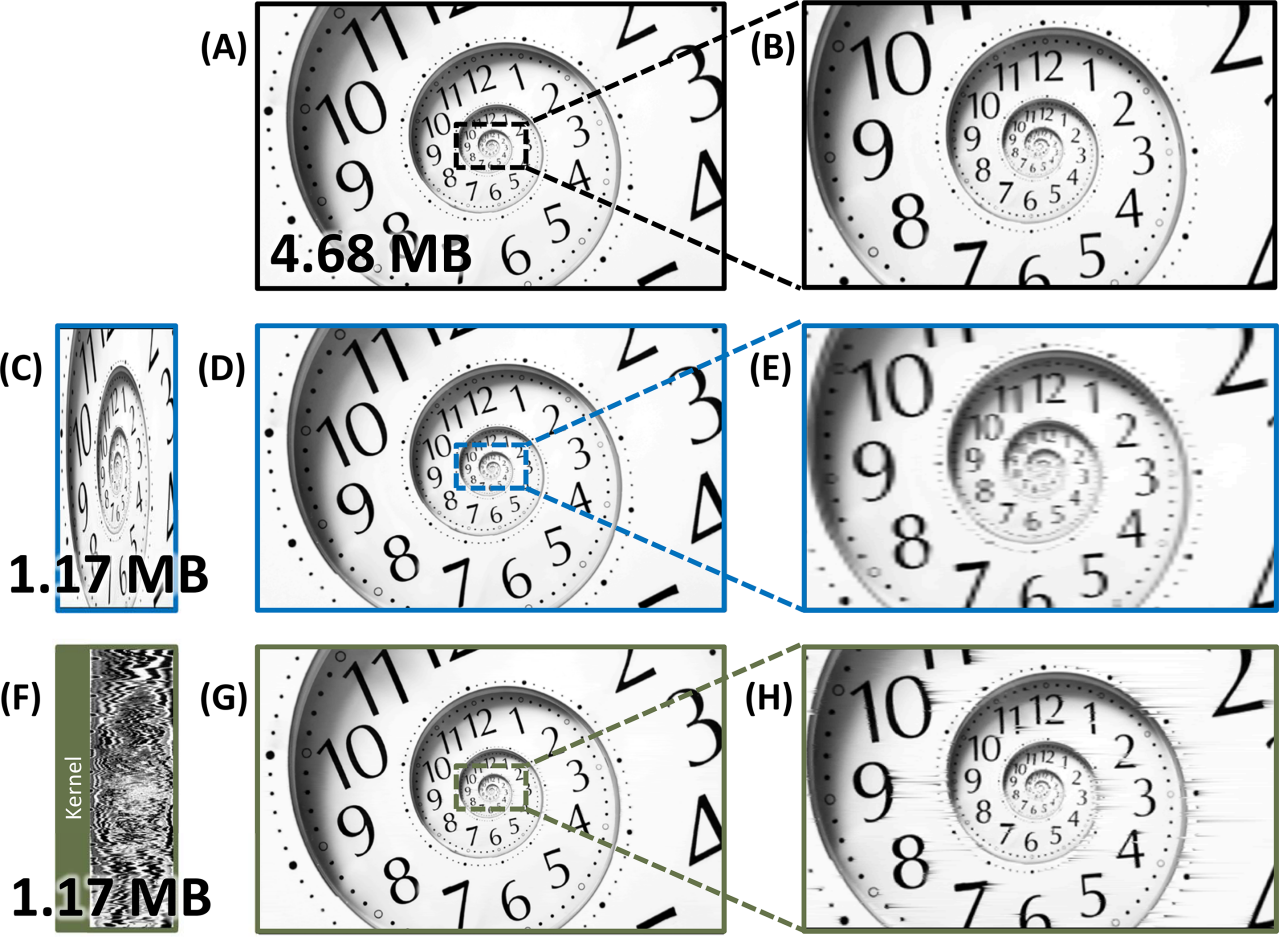
Comparison of compression performance with the fractal clock image. The original input image (A-B) and the 4X uniformly downsampled case (C-E), as compared with the reconstructed image after 5.25X warped stretch compression (F-H). The downsampling rate for the uniform case was increased (hence the image quality improvement) such that the resultant file sizes for both warped and uniform compression become equal (to compensate for the warp kernel overhead). After reconstruction, the warped case (G-H) achieved a PSNR of 37.7 dB, which was 6.32 dB better than the uniform downsampling case (D-E).

Fig 5 compares the reconstructed fractal clock images from the warped stretch compression and the uniformly downsampled compression methods at an overall compression ratio of 4X and 5.3X compression, respectively. Even after the rate in the uniform downsampling case was decreased to account for not having to save any kernel (which improved its output quality), the warped stretch image (Fig 5H) still significantly outperforms the uniformly downsampled image (Fig 5E). This is confirmed by an overall PSNR improvement of 6.32 dB.

**Fig 6:**
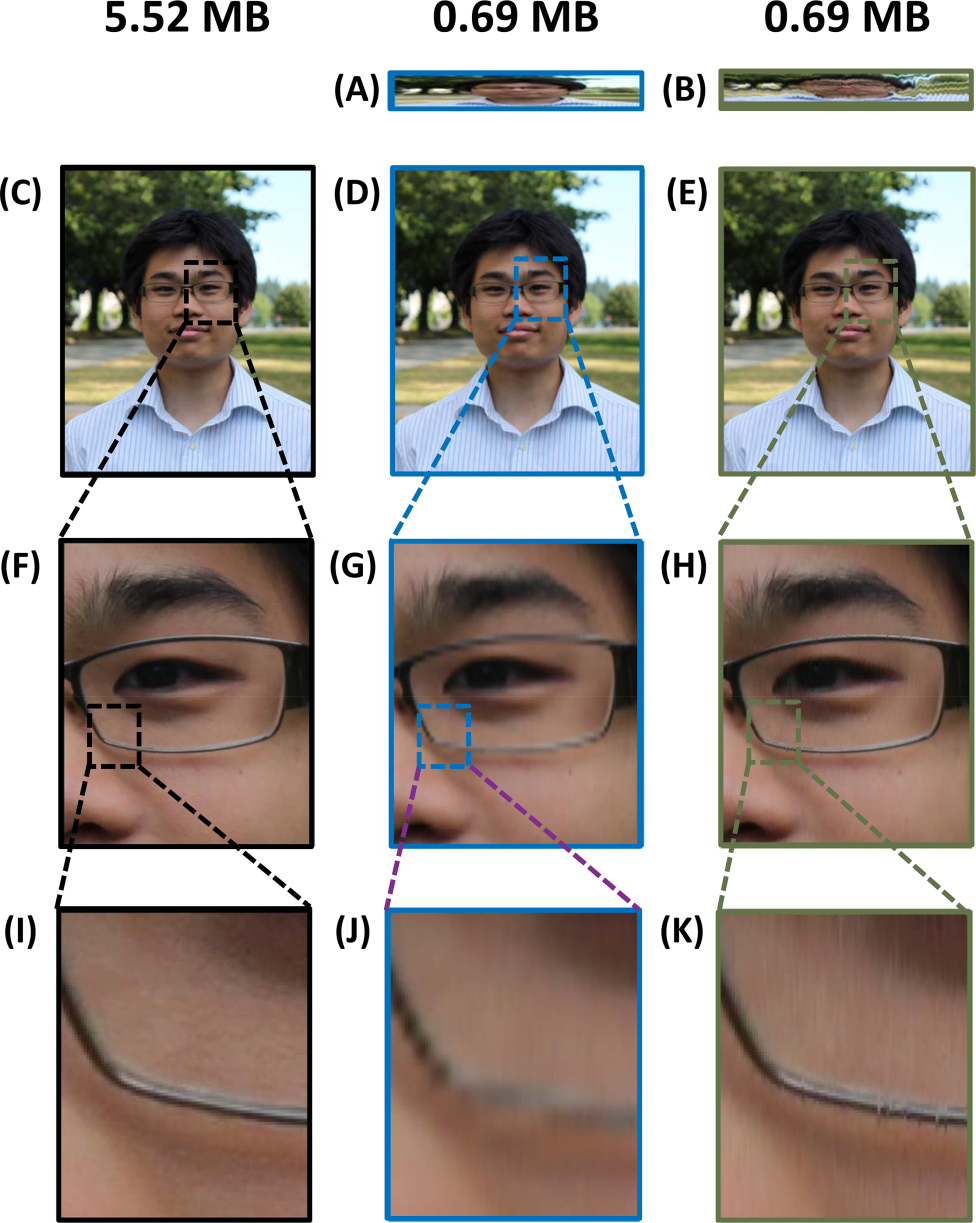
Comparison of compression performance with the colour portrait image. (A) The 8X uniformly downsampled image and (B) the 10.2X warp stretch-compressed image are shown with (C) the original image and (D-E) their respective reconstructions, while (F-H) are, in turn, their respective close-up portions. Further zoom-ins on the rims of the glasses are shown in (I-K), highlighting the failure of uniform downsampling to capture this sharp feature. The downsampling rate for the uniform case was adjusted such that the resultant file sizes for both warped and uniform compression become equal; however, since all three colour channels share the same warp kernel, the overhead burden is reduced by a third in this scenario. After reconstruction, the warped case (E,H,K) achieved a PSNR of 39.1 dB, which was 3.11 dB better than the uniform downsampling case (D,G,I).

We also performed warped stretch compression on a 3-channel RGB colour portrait image. Fig 6 shows the comparison in compression performance between uniform downsampling and warped stretch compression at 8X and 10X compression, respectively. All three channels here are able to share the same warp kernel, which reduces the metadata overhead. The warp kernel was generated using only the blue channel as it was the most feature-dense. The reconstruction performance exceeds the uniform downsampling case by approximately 3 dB at 10X compression.

**Fig 7:**
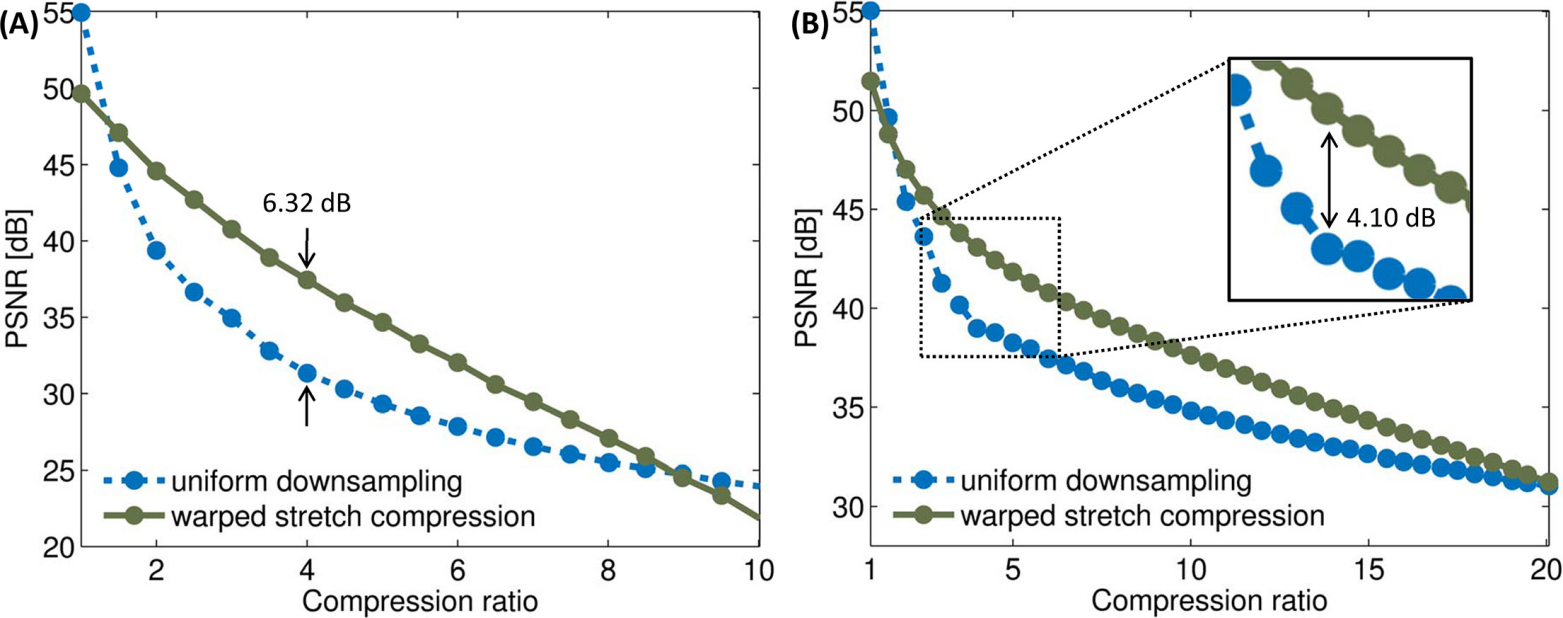
Empirical rate distortion plot for fractal clock and portrait images. The PSNR of warped stretch compression (solid) is compared with uniform downsampling (dotted) over a range of compression ratios for (A) the grayscale fractal clock image and (B) the colour portrait image. At a compression ratio of 4X, warped stretch outperforms in PSNR by 6.32 dB in the clock, and by 4.10 dB in the portrait. Beyond the compression ratios of 9X and 20X respectively, the overhead from the warp kernel completely compromises the performance.

Fig 7 shows the rate distortion plot for warped stretch compression as compared to compression with uniform downsampling for the fractal clock and the portrait images. Warped stretch compression was found to be superior to uniform downsampling from a compression ratio of 1.5X, up to 9X for the fractal clock and up to 20X for the portrait image. The range is extended for the colour image due to the lower overhead from the sharing of the warp kernel between colour channels. At a compression ratio of 4X, we find that warped stretch compression is better by more than 6 dB in PSNR for the grayscale clock and more than 4 dB for the color portrait. The lower PSNR difference for the colour image relative to the grayscale image can be attributed to the imperfect entropy redistribution in the red and green channels via using the warp kernel of the blue channel; the blue channel alone achieves a maximum PSNR improvement of 5.85 dB at 4X compression, as compared to 2.55 dB for the red channel, and 3.68 dB for the green channel at the same compression ratio.

To explain the rate-distortion behaviour, we consider how the same warp kernel is used for different compression ratios. As the compression ratio is increased, the post-warp uniform downsampling rate decreases, and the Nyquist condition is no longer satisfied. This is the main source of information loss in any compression scheme which involves downsampling.

## Conclusion

We have formulated a new type of digital image compression inspired by the recently demonstrated analog optical image compression enabled by warped stretch transform [36]. Our optics-inspired method warps the input image based on the distribution of its features, causing context-aware redistribution of the local entropy. Compared to the Fourier domain implementation of warped stretch image compression (DAST) reported earlier, this direct warping eliminates the need for phase retrieval in reconstruction. In this work, we limited the treatment to one-dimensional lines and simplify the analysis. We have shown more than 6 dB improvement in PSNR at a 4X compression ratio compared to the case of uniform downsampling. Future works would extend this to a full two-dimensional warped stretch transformation, and would investigate the combination of it with JPEG compression.
