# Correction: Context-Aware Image Compression

**DOI:** 10.1371/journal.pone.0168630

**Published:** 2016-12-12

**Authors:** The PLOS ONE Staff

Fig 1 appears incorrectly in the published article. Please see the correct Fig 1 and its caption here. The publisher apologizes for the error.

**Fig 1:**
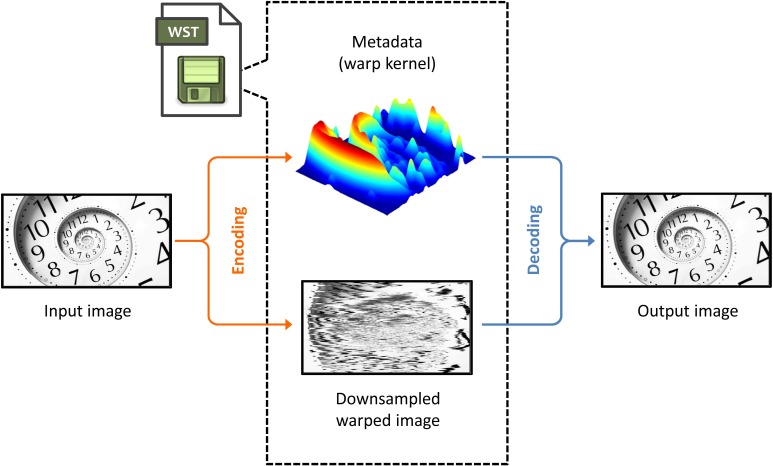
Overview schematic for image compression codec with warped stretch. The input is split into two components: i) the downsampled warped image and ii) the metadata, which contains a compressed version of the warp kernel. These two components are jointly used for recovering the original input. Since the warp kernel is image-dependent, we must send it as part of the compressed file, which creates extra overhead relative to an image-independent compression technique, such as uniform sampling. However, if the metadata can be compressed extremely compactly, the overall compression ratio can still be significant.
